# Central Nervous System Toxicity After Botulinum Neurotoxin Injection

**DOI:** 10.5812/aapm.9356

**Published:** 2013-07-01

**Authors:** Yashar Ilkhchoui, Ramsis F. Ghaly, N. Nick Knezevic, Kenneth D Candido

**Affiliations:** 1 Department of Anesthesiology and Critical Care Medicine, School of Medicine, University of New Mexico, Albuquerque, USA; 2 Department of Anesthesiology and Pain Management, Advocate Illinois Masonic Medical Center, Chicago, USA

**Keywords:** Botulinum Toxins, Type A, Parkinson Disease, Pain, Dystonic Disorders

## Abstract

Since Its first description of botulism toxicity in 1820s, specific formulations of botulinum neurotoxin (BoNT) have been introduced with different clinical benefits. However, there is increasing number of adverse events reported to Food and Drug Administration. This report presents the case of 62-year-old woman with Parkinson’s disease who received BoNT injections to treat painful spasticity in her hands. She developed severe generalized dystonia shortly after BoNT injections.

## 1. Introduction

Botulinum neurotoxin (BoNT) has been well studied in dermatology, neurology and other disciplines. The effectiveness of BoNT arises from its ability to inhibit the exocytosis of acetylcholine at peripheral cholinergic, motor, and autonomic nerve terminals. This causes a chemical denervation which results in partial to complete muscle paralysis. As its muscle relaxant effect is exerted in the hypertonic muscle, BoNT offers an alternative treatment for spastic patients who have difficulty with oral muscle relaxants that can produce generalized weakness, drowsiness, and cognitive impairment. In the past 20 years, BoNT has been used for a wide spectrum of Food and Drug Administration (FDA) approved disorders including strabismus, hemifacial spasm, blepharospasm, primary axillary hyperhidrosis and migraine headaches. There is growing number of off-label (not approved by FDA) therapeutic and cosmetic indications. Cerebral palsy related spasticities, poststroke spasticity, and piriformis syndrome are some of disorders that BoNT has been reported as a treatment option. Although commercially available preparations of BoNT have an excellent safety profile, especially for cosmetic purposes, there is expanding number of adverse events reported to FDA. We present a 62 year-old woman with Parkinson’s disease who received BoNT injections to treat painful spasticity in her hands. She developed severe generalized dystonia shortly after BoNT injections and extensive work-up did not reveal a definite etiology. We hypothesize that BoNT injection may have a causative role in her case.

## 2. Case Presentation

We present a 62 year-old female with Parkinson’s disease who was referred to the pain clinic for evaluation and treatment of painful contractures in her hands. She was diagnosed as having Parkinson’s disease 20 years ago when she initially presented with resting tremor. Her most recent drug regimen included amantadine, carbidopa-levodopa, and pramipexole without any change in the past 3 months. She stated that her painful contractures started nearly a year ago, had been worsening over time with no response to oral medications. She had bilateral hand contractures and deformities which involved mainly metacarpophalangial and proximal interphalangial joints. No specific pathological process other than Parkinson’s disease was identified to explain her chronic painful contractures. BoNT was introduced to her as a treatment option and she received BoNT type A (Botox; Allergan, Irvine, CA) injections in her hands targeting flexor digitorum profundus, flexor policis longus, adductor policis, and abductor policis brevis muscles. A dose of 50 units on each session was injected and she reported temporary relief of spasticity and pain. Two weeks after last session (total of four sessions with three-month intervals), she developed generalized dystonia presenting as severe neck torticollis, contractures of paravertebral muscles, and dystonic contractures of upper and lower limbs. She was unable to stand without aid; neurological exam was also significant for a resting tremor. Cranial nerves were intact, speech was fluent and clear, visual field and acuity were within normal limits. Deep tendon reflexes could not be elicited because of dystonia. Magnetic resonance imaging (MRI) of her brain did not reveal any abnormal finding.

## 3. Discussion

Clostridium botulinum, a gram-positive anaerobic bacterium, produces a toxin known as botulinum neurotoxin (BoNT). There are seven serotypes of BoNT: A, B, C (C1 and C2), D, E, F and G. BoNT A is currently approved by FDA as a treatment for cervical dystonia, blepharospasm, strabismus, upper limb spasticity, hyperhydrosis, and chronic migraines (1-4). BoNT-A is a large molecule with a molecular weight of 900 kDa, comprising a single chain with a heavy and light chain. Upon injection of BoNT-A, the molecule migrates toward the nerve terminal and attaches to it via the C portion of the heavy chain. Endocytosis occurs, causing the BoNT-A vesicle to be internalized and enter the nerve terminal. Next, the light chain portion of the BoNT-A detaches from the molecular vesicle and enters the cytoplasm. The light chain targets and cleaves the SNAP-25 (synaptosome-associated protein) of the SNARE complex, resulting in inhibition of acetylcholine release. This in turn causes decreased muscular contraction (5, 6). In addition, it is hypothesized that substance P and CGRP (calcitonin gene-related peptide) release are inhibited at the sensory afferent nerve. Glutamate, an excitatory neurotransmitter at the presynaptic membrane, is also inhibited. The inhibition of glutamate in turn further decreases the release of substance P and CGRP. Wide dynamic neuron activity is believed to be diminished, thus interrupting the overall sensory feedback mechanism. The inhibition of these neuropeptides involved in pain transmission and the overall interruption in the sensory feedback mechanism is hypothesized to be the cause of antinociceptive effects in chronic pain and ultimately the reduction of both peripheral and central sensitization (3, 7, 8). Since its initial use for strabismus treatment, BoNT injections have gained increasingly popularity as a therapeutic modality for the treatment of neurologic and non-neurologic conditions. Onset of paralysis can occur as quickly as six hours after administration, with clinical effects evident within 24 to 72 hours. Paralysis usually peaks two weeks after administration, followed by approximately 10 subsequent weeks of stable results. In the absence of additional doses, the muscle returns to full function on the order of 90 days. There are randomized, double-blind, placebo controlled studies evaluated efficacy of BoNT for the treatment of upper limb spasticity in post-stroke patients. BoNT effectively reduced the muscle tone compared to placebo but did not improve pain or range of motion and had minimal effect on functional disability (9, 10). However, BoNT demonstrated a significant improvement of pain in patients with cervical dystonia. It is generally believed that the adverse effects of BoNT are directly related to dosage. The most common adverse effects include pain, tenderness, erythema, and ecchymosis at the site of injection. Nonetheless, the principle concern when using BoNT is migration of the toxin from the target muscles to unintended structures resulting in systemic toxicity. Only one report in literature demonstrated the possibility that intramuscularly injected toxin might have induced a generalized botulism-like syndrome. However, this was a consequence of an undesired intravenous injection of BoNT, which partially escaped into venous circulation, rather than its diffusion out of the muscle into the systemic circulation (11). Although aspiration for blood before injection decreases the risk of venous injection, capillary uptake is likely to occur in some cases (12). Capillary uptake is more likely when using larger doses of toxin and/or larger volumes (13). Moreover, there are reports of BoNT capability to produce neutralizing antibodies in 5% of patients which decreases the effect of the toxin requiring higher doses and more frequent injections (14, 15). In the literature, there is only one study which evaluated in vivo central toxicity of BoNT in mice by injecting it into cerebrospinal fluid (CSF). Migration of the toxin from CSF to blood through the arachnoid villi causing systemic toxicity was suggested as a possible mechanism (16). FDA has reported adverse events after BoNT injection affecting nervous system far from initial site of injection such as speech disorder, nystagmus, restless leg syndrome, and even coma. Central nervous system involvement included 23.5% of serious and 24.9% of non-serious events (1). The mechanism of these adverse effects is not well understood, but it may represent an immunologically-mediated reaction to the foreign proteins in BoNT preparations (17). Careful attention to drug dose, dilution, site of injection is required for optimal outcome. Adverse events associated with the use of BoNT are significantly higher in patients with an underlying systemic disease. Some have suggested that using lower doses and more dilute concentrations may decrease the incidence of adverse effects in this patient population (8). Our patient presented with new symptoms totally different from her primary presentation as she developed generalized dystonia in contrast to focal spasticity in her hands. We hypothesized that BoNT can be absorbed by the systemic circulation even if injected intramuscularly and may result in idiosyncratic reactions in any organ-system distant from original site of injection including central nervous system.
